# Proteomic Study of Fetal Membrane: Inflammation-Triggered Proteolysis of Extracellular Matrix May Present a Pathogenic Pathway for Spontaneous Preterm Birth

**DOI:** 10.3389/fphys.2020.00800

**Published:** 2020-07-21

**Authors:** Jing Pan, Xiujuan Tian, Honglei Huang, Nanbert Zhong

**Affiliations:** ^1^Sanya Maternity and Child Care Hospital, Sanya, China; ^2^Proteomic Core Facility, Oxford University, Oxford, United Kingdom; ^3^New York State Institute for Basic Research in Developmental Disabilities, Staten Island, NY, United States

**Keywords:** fetal membrane, preterm birth, prelabor premature rupture of membrane, inflammation, extracellular matrix

## Abstract

**Introduction:**

Spontaneous preterm birth (sPTB), which predominantly presents as spontaneous preterm labor (sPTL) or prelabor premature rupture of membranes (PPROM), is a syndrome that accounts for 5–10% of live births annually. The long-term morbidity in surviving preterm infants is significantly higher than that in full-term neonates. The causes of sPTB are complex and not fully understood. Human placenta, the maternal and fetal interface, is an environmental core of fetal intrauterine life, mediates fetal oxygen exchange, nutrient uptake, and waste elimination and functions as an immune-defense organ. In this study, the molecular signature of preterm birth placenta was assessed and compared to full-term placenta by proteomic profiling.

**Materials and Methods:**

Four groups of fetal membranes (the amniochorionic membranes), with five cases in each group in the discovery study and 30 cases in each group for validation, were included: groups A: sPTL; B: PPROM; C: full-term birth (FTB); and D: full-term premature rupture of membrane (PROM). Fetal membranes were dissected and used for proteome quantification study. Maxquant and Perseus were used for protein quantitation and statistical analysis. Both fetal membranes and placental villi samples were used to validate proteomic discovery.

**Results:**

Proteomics analysis of fetal membranes identified 2,800 proteins across four groups. Sixty-two proteins show statistical differences between the preterm and full-term groups. Among these differentially expressed proteins are (1) proteins involved in inflammation (HPGD), T cell activation (PTPRC), macrophage activation (CAPG, CD14, and CD163), (2) cell adhesion (ICAM and ITGAM), (3) proteolysis (CTSG, ELANE, and MMP9), (4) antioxidant (MPO), (5) extracellular matrix (ECM) proteins (APMAP, COL4A1, LAMA2, LMNB1, LMNB2, FBLN2, and CSRP1) and (6) metabolism of glycolysis (PKM and ADPGK), fatty acid synthesis (ACOX1 and ACSL3), and energy biosynthesis (ATP6AP1 and CYBB).

**Conclusion:**

Our molecular signature study of preterm fetal membranes revealed inflammation as a major event, which is inconsistent with previous findings. Proteolysis may play an important role in fetal membrane rupture. Extracellular matrix s have been altered in preterm fetal membranes due to proteolysis. Metabolism was also altered in preterm fetal membranes. The molecular changes in the fetal membranes provided a significant molecular signature for PPROM in preterm syndrome.

## Introduction

Preterm birth refers to infants born alive before 37 weeks of gestation (Quinn et al., 2016). Preterm birth accounts for 5–12% of all live births worldwide (Gezer et al., 2018). As a populated country, China has about 1.17 million preterm infants birth every year (World Health Organization [WHO], 2018). In particular, since 2016, when the Chinese government relaxed the one-child policy and implemented its two-children policy, many women of high maternal age rushed to have a second child (Cheng and Duan, 2016). It has been demonstrated that pregnancy at 40 years of age and older is strongly associated with preterm birth and other disorders of pregnancy (Fuchs et al., 2018). In preterm newborns, the brain, lung, liver, and other organs are not fully developed, and there is a high incidence of brain injury, neonatal respiratory distress syndrome, bilirubin encephalopathy, and multiple organ failure (Fraser et al., 2004; Bhutani and Wong, 2013; Paton et al., 2017). It is estimated by the WHO that preterm birth is the most common cause of children’s death under the age of 5 years (World Health Organization [WHO], 2018). Preterm birth brings a great economic burden to families, communities, and societies.

The placenta serves as the interface between the pregnant mother and the intrauterine fetus, with the main function of exchanging the material between the fetus and the mother, providing the oxygen and nutrients required for embryonic, as well as fetal, development, and excreting metabolic waster and CO_2_ (Gude et al., 2004). In addition, the placenta also serves as a barrier to bacteria, pathogens, and drugs because of the existence of the placental barrier (Zeldovich et al., 2013). The placenta can also synthesize chorionic gonadotropin, human placental lactogen (hPL), estrogen, progesterone, cytokine, and growth factors (Costa, 2016). In addition, the placenta has immune tolerance to the fetus (Guleria and Sayegh, 2007). Therefore, abnormal placental function has a direct correlation with the occurrence of preterm birth. So far, estrogens, hPL, placenta growth factor (PLGF), human chorionic gonadotrophin (hCG), plasma protein A (PAPP-A), placental protein 13 (PP-13), pregnancy-specific glycoproteins, and progesterone metabolites have been employed as surrogate markers of placental function (Heazell et al., 2015). Despite recent progress on the study of these markers associated with function of placenta, a comprehensive and systemic understanding of the pathophysiology of the placenta is lacking, particularly little is known about the pathogenic role of fetal membrane and its involvement in the development of spontaneous preterm birth (sPTB). Here, we studied the protein expression of fetal membrane, with the aim to understand the pathophysiology of fetal membrane and to identify novel molecules associated with preterm birth in the fetal membrane. This study not only provides theoretical support for the occurrence of preterm birth, but also provides reference for the early diagnosis and early intervention of premature birth.

## Materials and Methods

### Specimens

The study was approved by the Hospital Ethics Committee of Sanya Maternity and Child Care Hospital and the informed consent was obtained from pregnant women to permit the use of placentas in research studies. A retrospective study was designed to investigate the pathological alteration of protein expression with a pre-banked birth cohort of 20 placentas, which were grouped as (A): spontaneous preterm labor (sPTL), defined as a non-medical and/or non-selective spontaneous birth delivered between 20^+1^ and 36^+6^ gestational week (GW) in which regular contractions of the uterus result in changes in the cervix before 37 weeks of pregnancy, (B): prelabor premature rupture of membranes (PPROM) that occurred between 20^+1^ and 36^+6^ GW, (C): full-term birth (FTB) between 39^+1^ and 40^+6^ GW, and (D): full-term premature rupture of membrane (PROM). There were five placentas in each group. The placentas were collected from fetal membranes through full layers to the decidua. Fetal membranes were dissected within 2 cm of the edge where the membrane naturally ruptured during labor. However, if a rupture hole could be identified in the premature rupture (PPROM and PROM) cases, the membrane would be collected within 1 cm around the hole where the premature rupture occurred.

### Criteria for Inclusion and Exclusion

Tissues selected from prebanked samples had to meet the following criteria: (i) age of the pregnant woman is 18–45 years, (ii) no clinically recognized infection/inflammation (INF) before and/or during pregnancy (INF is determined by phenotypically notable fever, increased counts of peripheral white blood cells, and/or increased IL6 and/or TNFα), (iii) primipara and singleton without history of miscarriage or abortion, (iv) vaginal delivery with (PPROM and PROM) or without (FTB and sPTL) premature rupture of chorioamniotic membrane, (v) no vaginal bleeding during the pregnancy, (vi) no other pregnancy-related complication(s) and no clinical intervention with antibiotics, steroids, or tocolytics during the pregnancy, (vii) no family history of birth defects, and (viii) no consanguinity. Any cases not meeting the above criteria were excluded. Details of demographic and clinical information for the samples studied are provided in Table 1.

**TABLE 1:** Demographic and clinical information about placentas.

Group	A	B	C	D
Birth	sPTL	PPROM	FTB	PROM
Pregnant age (year old)	22-28	22-30	22-25	22-26
Gestational age (weeks)	29^+3^–31^+6^	30^+0^–32^+1^	40^+0^–40^+6^	39^+0^–40^+6^
Primipara	Yes	Yes	Yes	Yes
Singleton	Yes	Yes	Yes	Yes
Mode of delivery	Spontaneous	Spontaneous	Spontaneous	Spontaneous
Family history of preterm birth	No	No	No	No
Family history of birth defect	No	No	No	No
Infection history during pregnancy	No	No	No	No
Gestational complication	No	No	No	No
Use of antibiotics during pregnancy	No	No	No	No
Use of steroid	No	No	No	No
Inform consent obtained	Yes	Yes	Yes	Yes

### Protein Extraction From Placenta Membrane for Proteomic Analysis

**FIGURE 1:**
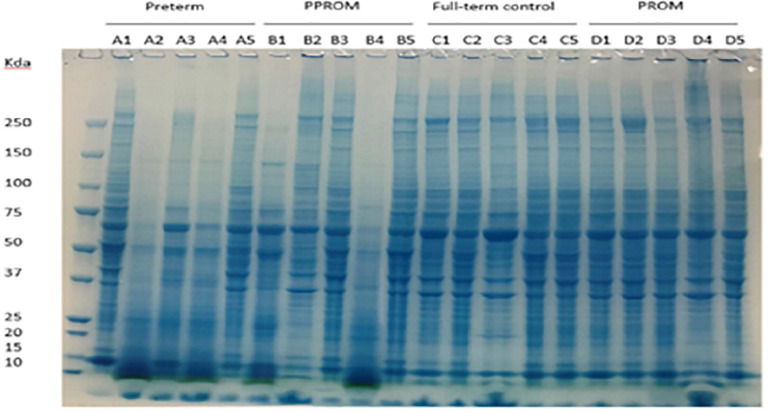
Protein expression pattern on SDS-PAGE gel from four groups of fetal membrane. Group A: sPTL (preterm), group B: PPROM, group C: FTB, and group D: PROM. Five individual samples in each group were analyzed on an 8% SDS-PAGE stained with a dye of Instantblue.

The human placental amniochorionic membrane, or fetal membrane, was dissected (20- to 30-mg cross-sections) and first washed with cold PBS containing protease inhibitor (Sigma-Aldrich, United Kingdom), then placed in beads-beater tubes containing RIPA lysis buffer (Thermo Fisher Scientific, United Kingdom) to make it 50 mg/ml. Tissues were homogenized four times at 6,500 Hz for 40 s in a beads-beater (Stretton, United Kingdom) (Figure 1). The samples were centrifuged at 10,000 g for 5 min at 4°C to remove insoluble tissue debris. The protein concentration in the homogenates was determined by BCA assay (Thermo Fisher Scientific, United Kingdom), and 100 μg of total proteins were added to a 30-kDa filter (Merck Millipore, United Kingdom). Proteins were reduced by 10 mM Dithiothreitol (DTT) (Sigma, United Kingdom) at 37°C for 1 h and then alkylated with 40 mM iodoacetamide (IAA, Sigma, United Kingdom) for 45 min in the dark, at room temperature. Samples were centrifuged for 20 min at 14,000 g to remove DTT and IAA, followed by buffer exchange with 8 M urea twice and 50 mM ammonia bicarbonate three times. One hundred microliters of trypsin were added at a trypsin/protein ratio of 1:50 for digestion at 37°C overnight. Digested peptides were collected by upside down spin, and membrane filters were washed twice with 0.5 M NaCl and water, respectively. The peptides were purified by a SepPak C18 cartridge (Waters, United Kingdom), dried by SpeedVac centrifugation, and resuspended in buffer A (2% acetonitrile, 0.1% formic acid) for LC-MS/MS analysis.

### Peptide Measurement by Mass Spectrometry

LC-MS/MS analysis was carried out by nano-ultra performance liquid chromatography tandem mass spectrometry analysis using a 75-μm-inner diameter × 25 cm C18 nanoAcquity UPLC column (1.7-μm particle size, Waters, United Kingdom). Peptides were separated with a 120-min gradient of 3–40% solvent B (solvent A: 99.9% H_2_O, 0.1% formic acid; solvent B: 99.9% ACN, 0.1% formic acid) at 250 nl/min and injected into a Q Exactive High Field (HF) Hybrid Quadrupole-Orbitrap Mass Spectrometer (Thermo Fisher Scientific, United Kingdom) acquiring data in electron spray ionization (ESI) positive mode. The MS survey was set with a resolution of 60,000 FWHM, with a recording window between 300 and 2,000 *m*/*z*. A maximum of 20 MS/MS scans were triggered in data-dependent acquisition (DDA) mode.

### Protein Identification and Quantification

MaxQuant software (v1.5.8.3, Max Planck Institute of Biochemistry, Germany) was used for peptide and protein identification and quantitation. Data generated from MS/MS spectra were searched against the Uniprot human database (version 2017); 20,205 entries were used for peptide homology identification. The false discovery rate (FDR) was set to 1% for protein and peptide identification. Proteins were quantified by at least one unique peptide, and match between run was selected to increase the quantifiable value cross samples (Figure 1). Label-free quantitation (LFQ) intensity data were used for further analysis and comparisons across the variant groups. Statistical analysis was assessed by using Persus software (version 1.5.5.3, Max Planck Institute of Biochemistry). Statistical comparisons between the groups were performed by using two-sided unpaired Student’s *t*-test. Firstly, threshold p-value was used to define statistical significance. Secondly, permutation-based FDR was used to assess truncate data.

### Validation of Differentially Expressed Proteins With Western Blot

Ten milligrams of fetal membranes or placental villi were dissected and lysed in RIPA buffer containing protease inhibitors (Roche, United States). Western blot analysis was performed by loading 15 μg of proteins on 4–12% pre-cast Bis-Tris gels (Bio-Rad, United States) and transferred to PVDF membranes (Merck Millipore, United States). Membranes were incubated with mouse anti-human MPO monoclonal antibody (Santa Cruz, United States, 1:200 diluted in 2% milk), mouse anti-human elastase, neutrophil expressed (ELANE) monoclonal antibody (Santa Cruz, United States, 1:200 diluted in 2% milk), mouse anti-human GAPDH monoclonal antibody (Thermo Fisher Scientific, United Kingdom, 1:2,000 diluted in 2% milk), rabbit anti-human beta-actin antibody (Abcam, United Kingdom, 1:1,000 diluted in 2% milk). Dye-800-conjugated secondary antibodies were applied and visualized with an Odyssey Clx (Li-Cor, United States). Image studio (Li-Cor, United States) and Image J (Schneider et al., 2012) were used for Western blot quantitation, and one-way ANOVA was used for statistical significance test.

## Results

### Quality Control of Proteins Isolated From the Fetal Membrane Tissues

Protein lysates of fetal membranes were run on SDS-PAGE and visualized with InstantBlue (Figure 1). Protein partial degradation was detected in samples of A2, A4 and B1, B4. The protein patterns of the rest of samples did not show a clear difference.

### Differentially Expressed Proteins Identified From Mass Spectra

**FIGURE 2:**
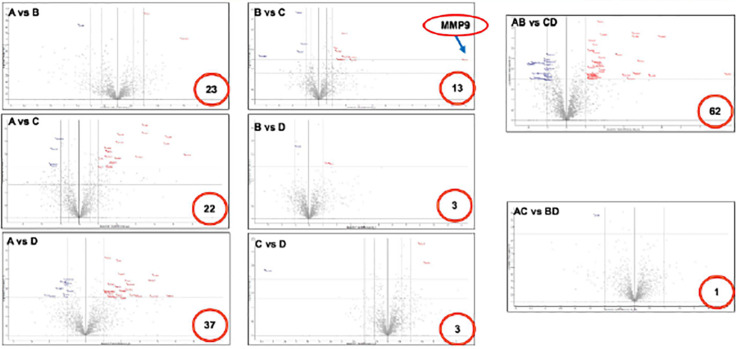
Volcano plot of differentially expressed proteins in fetal membranes. Various comparisons identified specific protein(s) that associated with the premature condition. Number in the red circle indicates the number of differential proteins with statistical significance. *X*-axis indicates fold changes and *Y*-axis indicates *p* value-log Student *t*-test. A: sPTL, B: PPROM, C: FTB, and D: PROM.

After LC-MS/MS measurement, Maxquant analysis identified a total of 2,880 proteins from fetal membrane samples. The hierarchical clustering analysis indicated protein abundance alteration between individual samples. In addition to comparisons of the preterm group (A + B) with the full-term control group (C + D), individual comparisons were applied to identified specific protein(s) that are associated with a specific condition (Figure 2). Protein differential expression was defined by the criteria of *p* < 0.01, fold change (FC) ≥ 2, and permutation-based FDR 0.05, with which, 62 (38 up-regulated and 24 down-regulated) proteins were identified to be differentially expressed (Tables 2a,b). Among these 62 proteins, 20 were identified to be the top-listed FC (four were down-regulated and 16 were up-regulated). The FCs of 8 of 20 up-regulated proteins were >11, among which MMP9 showed 318.64 FC as the highest (Table 3). All differentially expressed proteins identified from fetal membranes in sPTB (sPTL and PPROM) were subjected to Kyoto Encyclopedia of Genes and Genomes (KEGG) analysis to identify the pathways that may be associated with the sPTB. The top-scoring pathways were infection and inflammation, protein degradation and proteolysis, extracellular matrix (ECM), cell adhesion, antioxidant, glycolysis, and fatty acid (FA) oxidation (Table 4).

**TABLE 2a:** Proteins identified from fetal membranes associated with preterm birth (AB vs. CD): up-regulated.

Majority protein IDs	Protein names	Gene names	Protein family	Pathways	Functions
Q9HDC9; H0Y512; Q9HDC9-2	Adipocyte plasma membrane-associated protein	APMAP	Adipocyte plasma membrane-associated protein (PTHR10426:SF26)		
Q15904; A0A0C4DGX8	V-type proton ATPase subunit S1	ATP6AP1	V-type proton ATPase subunit S1 (PTHR12471:SF2)	Energy metabolism	ATP synthase
Q93050-1; Q93050; Q93050-3; B7Z641; B7Z2A9; F5H1T6	V-type proton ATPase 116 kDa subunit a isoform 1; V-type proton ATPase subunit a	ATP6V0A1	V-type proton ATPase 116 kDa subunit a isoform 1 (PTHR11629:SF68)	Energy metabolism	ATP synthase
F5GYQ1; P61421; J3QL14; R4GN72	V-type proton ATPase subunit d 1	ATP6V0D1	V-type proton ATPase subunit d 1 (PTHR11028:SF3)	Energy metabolism	ATP synthase
A6NC48; Q10588; H0Y984; Q10588-2	ADP-ribosyl cyclase/cyclic ADP-ribose hydrolase 2	BST1	ADP-ribosyl cyclase/cyclic ADP-ribose hydrolase 2 (PTHR10912:SF4)		
A0A0A0MSV6; D6R934; P02746; D6RGJ1	Complement C1q subcomponent subunit B	C1QB	Complement C1q subcomponent subunit B (PTHR44403:SF2)		NAD metabolism and Innate Immune System
B4DPQ0; P00736; F5H2D0	Complement C1r subcomponent; Complement C1r subcomponent heavy chain; Complement C1r subcomponent light chain	C1R	Complement C1r subcomponent (PTHR45206:SF1)		
P08571; D6RFL4	Monocyte differentiation antigen CD14; Monocyte differentiation antigen CD14, urinary form; Monocyte differentiation antigen CD14, membrane-bound form	CD14	Monocyte differentiation antigen CD14 (PTHR10630:SF3)	Toll receptor signaling pathway (P00054)	
F5GZZ9; Q86VB7-3; Q86VB7; Q86VB7-2; C9JHR8; Q86VB7-4	Scavenger receptor cysteine-rich type 1 protein M130; Soluble CD163	CD163	Scavenger receptor cysteine-rich type 1 protein M130 (PTHR19331:SF392)	Macrophages function	Acute phase-regulated receptor involved in clearance and endocytosis of hemoglobin/haptoglobin complexes by macrophages
H0YD13; P16070-18; P16070-12; P16070-14; P16070-13; P16070-11; P16070-10; P16070-16; P16070-8; P16070-17; P16070-6; P16070-4; P16070-3; P16070-7; P16070-5; P16070; H0Y2P0; H0YE40	CD44 antigen	CD44	CD44 antigen (PTHR10225:SF6)		
F8VNT9; F8VV56; F8W022; F8VWK8; P08962-3; P08962-2; P08962	Tetraspanin; CD63 antigen	CD63	CD63 antigen (PTHR19282:SF233)		
P02452	Collagen alpha-1 (I) chain	COL1A1	Collagen alpha-1(I) chain (PTHR24023:SF569)	Integrin signaling pathway (P00034)	Extracellular matrix structural constituent
P02462-2; P02462	Collagen alpha-1(IV) chain; Arresten	COL4A1	Collagen alpha-1(IV) chain (PTHR24023:SF854)	Integrin signaling pathway (P00034)	Extracellular matrix structural constituent
P08311	Cathepsin G	CTSG	Cathepsin G (PTHR24271:SF13)	Protein degradation	Proteolysis
P04839	Cytochrome b-245 heavy chain	CYBB	Cytochrome b-245 heavy chain (PTHR11972:SF60)	Energy metabolism	ATP synthase/oxidase
P08246	Neutrophil elastase	ELANE	Neutrophil elastase (PTHR24257:SF16)	Protein degradation	Proteolysis/phagocytosis
A0A0D9SEN1;Q12884; B4DLR2	Prolyl endopeptidase FAP; Antiplasmin-cleaving enzyme FAP, soluble form	FAP	Prolyl endopeptidase FAP (PTHR11731:SF136)	UPS	Ubiquitin-protein ligase activity (ubiquitin proteasome system)
P98095-2; P98095	Fibulin-2	FBLN2	Fibulin-2 (PTHR44887:SF1)		
Q86UX7-2; Q86UX7;F5H1C6	Fermitin family homolog 3	FERMT3	Fermitin family homolog 3 (PTHR16160:SF1)		
P02792	Ferritin light chain	FTL	Ferritin light chain (PTHR11431:SF47)		
P11413; P11413-3; P11413-2; E9PD92; E7EM57; E7EUI8	Glucose-6-phosphate 1-dehydrogenase	G6PD	Glucose-6-phosphate 1-dehydrogenase (PTHR23429:SF0)	Glycolysis	Glycolysis
P05204; A0A087WZE9; Q15651-2; Q15651	Non-histone chromosomal protein HMG-17; High mobility group nucleosome-binding domain-containing protein 3	HMGN2;HMGN3			
P05362; K7EKL8	Intercellular adhesion molecule 1	ICAM1	Intercellular adhesion molecule 1 (PTHR13771:SF9)		Cell adhesion signaling
P11215; P11215-2	Integrin alpha-M	ITGAM	Integrin alpha-M (PTHR23220:SF120)	Inflammation mediated by chemokine and cytokine signaling pathway	
(P00031)/Integrin signaling pathway (P00034)					
A0A087WX80; P24043; A0A087WYF1	Laminin subunit alpha-2	LAMA2	Laminin subunit alpha-2 (PTHR10574:SF291)	Integrin signaling pathway (P00034)	Extracellular matrix linker protein receptor
P20700; E9PBF6; A0A0D9SFE5	Lamin-B1	LMNB1	Lamin-B1 (PTHR23239:SF157)	FAS signaling pathway (P00020)	Structual molecule activity
Q03252	Lamin-B2	LMNB2	Lamin-B2 (PTHR23239:SF152)	FAS signaling pathway (P00020)	Structual molecule activity
P14780	Matrix metalloproteinase-9; 67 kDa matrix metalloproteinase-9; 82 kDa matrix metalloproteinase-9	MMP9	Matrix metalloproteinase-9 (PTHR10201:SF30)	Plasminogen activating cascade (P00050)/CCKR signaling(P06959)	Collagenases (degrade collagen)
P05164-2; P05164; P05164-3	Myeloperoxidase; Myeloperoxidase; 89 kDa myeloperoxidase; 84 kDa myeloperoxidase; Myeloperoxidase light chain; Myeloperoxidase heavy chain	MPO	Myeloperoxidase (PTHR11475:SF108)		Antioxidant (GO:0016209)
Q8IXM6; H0Y6T6; Q8IXM6-2	Nurim	NRM	Nurim (PTHR31040:SF1)		
Q14980-2; Q14980; A0A087WY61; Q14980-4; Q14980-3; Q14980-5	Nuclear mitotic apparatus protein 1	NUMA1	Nuclear mitotic apparatus protein 1 (PTHR18902:SF24)		
Q03405; Q03405-3; M0R0Y4; M0QYR6; M0R1I2; Q03405-2	Urokinase plasminogen activator surface receptor	PLAUR	Urokinase plasminogen activator surface receptor (PTHR10624:SF6)	Plasminogen activating cascade (P00050)/Blood coagulation (P00011)	
A2ACR1; P28065-2; P28065; A0A0G2JJA7; A2ACR0	Proteasome subunit beta type; Proteasome subunit beta type-9	PSMB9	Proteasome subunit beta type-9 (PTHR11599:SF50)	UPS	Ubiquitin proteasome system
X6R433; A0A0A0MT22; P08575-2; P08575; M3ZCP1; A0A075B788; E9PKH0	Protein-tyrosine-phosphatase; Receptor-type tyrosine-protein phosphatase C	PTPRC	Receptor-type tyrosine-protein phosphatase C (PTHR19134:SF284)	JAK/STAT signaling pathway (P00038)/B cell activation (P00010)/T cell activation (P00053)	T cell activation (P00053)
P05109	Protein S100-A8; Protein S100-A8, N-terminally processed	S100A8	Protein S100-A8 (PTHR11639:SF5)		Calmodulin signaling
P06702	Protein S100-A9	S100A9	Protein S100-A9 (PTHR11639:SF79)		Calmodulin signaling
P01011; G3V595; G3V3A0	Alpha-1-antichymotrypsin; Alpha-1-antichymotrypsin His-Pro-less	SERPINA3	Alpha-1-antichymotrypsin (PTHR11461:SF145)		Proteolysis (inhibitor)
A0A0C4DFU2; P04179; P04179-4; F5H4R2; A0A0C4DFU1; F5GYZ5; P04179-2; F5H3C5; G8JLJ2; A0A0C4DG56; P04179-3	Superoxide dismutase;Superoxide dismutase [Mn], mitochondrial	SOD2	Superoxide dismutase [Mn], mitochondrial (PTHR11404:SF6)		Antioxidant defense activity

**TABLE 2b:** Proteins identified from fetal membranes associated with preterm birth (AB vs. CD): Down-regulated.

Majority protein IDs	Protein names	Gene names	Protein family	Pathways	Functions
Q15067-2; Q15067; Q15067-3	Peroxisomal acyl-coenzyme A oxidase 1	ACOX1	Peroxisomal acyl-coenzyme A oxidase 1 (PTHR10909:SF290)	Fatty acid metabolism	Fatty acid beta-oxidation
095573	Long-chain-fatty-acid–CoA ligase 3	ACSL3	Long-chain-fatty-acid–CoA ligase 3 (PTHR43272:SF13)	Fatty acid metabolism	Fatty acid metabolic process
P09525; Q6P452	Annexin A4; Annexin	ANXA4	Annexin A4 (PTHR10502:SF28)		
P40121; P40121-2; E7ENU9	Macrophage-capping protein	CAPG	Macrophage-capping protein (PTHR11977:SF13)	FAS signaling pathway (P00020)	Macrophage function/Structual molecule activity
P21291; E9PS42; E9PND2; E9PP21	Cysteine and glycine-rich protein 1	CSRP1	Cysteine and glycine-rich protein 1 (PTHR24215:SF23)		Structual molecule activity
Q9UHQ9; H7C0R7	NADH-cytochrome b5 reductase 1	CYB5R1	NADH-cytochrome b5 reductase 1 (PTHR19370:SF74)		Oxidoreductase activity
P05108; P05108-2; E7EPP8	Cholesterol side-chain cleavage enzyme, mitochondrial	CYP11A1	Cholesterol side-chain cleavage enzyme, mitochondrial (PTHR24279:SF3)	Androgen/estrogene/progesterone biosynthesis (P02727)	
E7EQR4; P15311	Ezrin	EZR	Ezrin (PTHR23281:SF13)		Structual molecule activity
P15428; P15428-5; P15428-2; E9PBZ2; P15428-4	15-hydroxyprostaglandin dehydrogenase [NAD(+)]	HPGD	15-hydroxyprostaglandin dehydrogenase [NAD(+)] (PTHR44229:SF4)		
P40925; P40925-3; B9A041; P40925-2; B8ZZ51	Malate dehydrogenase, cytoplasmic	MDH1	Malate dehydrogenase, cytoplasmic (PTHR23382:SF3)	TCA cycle (P00051)	
E9PIY1; A0A0C4DGG1; Q9UKS6; E9PJ75; E9PNM9	Protein kinase C and casein kinase substrate in neurons protein 3	PACSIN3	Protein kinase C and casein kinase substrate in neurons protein 3 (PTHR23065:SF18)		
P30086	Phosphatidylethanolamine-binding protein 1; Hippocampal cholinergic neurostimulating peptide	PEBP1	Phosphatidylethanolamine-binding protein 1 (PTHR11362:SF28)	EGF/FGF receptor signaling pathway (P00018)	
P14618; P14618-3; B4DNK4	Pyruvate kinase PKM; Pyruvate kinase	PKM	Pyruvate kinase PKM (PTHR11817:SF15)	Glycolysis (P00024)	
P30044-2; P30044; P30044-3; P30044-4	Peroxiredoxin-5, mitochondrial	PRDX5	Peroxiredoxin-5, mitochondrial (PTHR10430:SF16)		
P61313; E7EQV9; E7ENU7; E7EX53; P61313-2	60S ribosomal protein L15; Ribosomal protein L15	RPL15	60S ribosomal protein L15 (PTHR11847:SF13)		
P62888; E5RI99; A0A0B4J213; A0A0C4DH44	60S ribosomal protein L30	RPL30	60S ribosomal protein L30 (PTHR11449:SF1)		Biosynthetic process
P46777	60S ribosomal protein L5	RPL5	60S ribosomal protein L5 (PTHR23410:SF12)		Biosynthetic process
P62277; J3KMX5	40S ribosomal protein S13	RPS13	40S ribosomal protein S13 (PTHR11885:SF6)		Biosynthetic process
P62249; M0R210; A0A087WZ27; M0R3H0; M0R1M5	40S ribosomal protein S16	RPS16;ZNF90			Biosynthetic process

**TABLE 3:** Top-20 proteins identified from fetal membranes associated with preterm birth.

Gene symbol	Protein name	Function	*p*-value	Fold change
CSRP1	Cysteine and glycine-rich protein 1	Extracellular metrix	1.75E-03	–4.11
HPGD	15-hydroxyprostaglandin dehydrogenase [NAD (+)]	Inflammation	1.70E-03	–3.95
PKM	Pyruvate kinase	Glycolysis	1.46E-03	–3.59
ACSL3	Long-chain-fatty-acid-CoA ligase 3	Fatty acid metabolism	1.45E-03	–3.45
FBLN2	Fibulin-2	Extracellular metrix	1.36E-03	2.81
HMGN2	High mobility group nucleosomal binding domain 2	Gene transcription	2.19E-03	3.05
HMGN3	High mobility group nucleosomal binding domain 3	Gene transcription	2.19E-03	3.05
CD14	Monocyte differentiation antigen CD14	Macrophage function	1.49E-05	3.14
BST1	ADP-ribosyl cyclase/cyclic ADP-ribose hydrolase 2	B-cell growth	1.92E-03	3.25
CD163	Scavenger receptor cysteine-rich type 1 protein M130	Macrophage function	8.10E-04	3.29
LMNB1	Lamin-B1	Extracellular metrix	6.31E-05	3.47
ICAM1	Intercellular adhesion molecule 1	Cell adhesion	5.86E-04	6.07
PTPRC	Receptor-type tyrosine-protein phosphatase C	T cell activation	5.36E-05	11.24
CYBB	Cytochrome b-245 heavy chain	Energy metabolism	7.48E-05	11.24
S100A9	Protein S100-A9	Calmodulin signaling	4.82E-03	11.47
MPO	Myeloperoxidase	Oxidative stress	5.18E-03	11.99
CTSG	Cathepsin G	Protein degradation	1.22E-03	13.56
ELANE	Neutrophil elastase	Protein degradation	6.02E-03	20.22
ITGAM	Integrin alpha-M	Cell adhesion	7.61E-05	28.71
MMP9	Matrix metalloproteinase-9	Protein degradation	5.25E-03	318.64

**TABLE 4:** Proteins involved in top-scored pathways in fetal membrane of sPTB.

Pathway	Up-regulated protein	Down-regulated protein
Infection and inflammation	PTPRC, BST1, CAPG, CD14, CD163, S100A9	HPGD, S100P
Protein degradation and proteolysis	CTSG, ELANE, MMP9	
Extracellular matrix	APMAP, COL4A1, LAMA2, LMNB1, LMNB2, FBLN2	
Cell adhesion	ICAM1, ITGAM	CSRP1
Antioxidant	MPO	
Glycolysis		PKM
Fatty acid beta-oxidation		ACOX1, ACSL3

### Validation of Differentially Expressed Proteins With Western Blots

**FIGURE 3:**
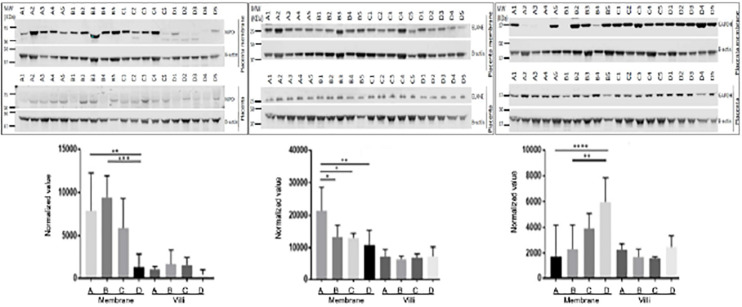
Validation of differentially expressed proteins with placenta villi and fetal membranes. Randomly selected proteins MPO **(left panel)**, ELANE **(middle panel)**, and GAPDH **(right panel)** that were identified to be differentially expressed in fetal membrane (FM) vs. placental villi (PV) by MS/MS were validated with Western blots. Generally, three proteins were up-regulated in fetal membranes when compared to placental villi. MPO and ELANE were significantly increased in preterm pregnancies of sPTL (A) & PPROM (B) compared to full-term pregnancies of FTB (C) and PROM (D) fetal membranes. However, GAPDH was decreased in A + B. *p < 0.05, **p < 0.01, ***p < 0.005, and ****p < 0.0001. Beta-Actin was used as an internal control for normalizing the protein expression value.

Three proteins, MPO, ELANE, and GAPDH, which were shown by LC-MS/MS to be differentially expressed in fetal membrane, were randomly selected to validate the differential expression value of protein with Western blots. Westerns blot of MPO and ELANE showed the same protein expression pattern as proteome data with statistical significance. While GAPDH followed a similar increase of protein expression in fetal membrane compared to placental villi, it had a decreased expression in fetal membrane of preterm cases, including both sPTL and PPROM, compared to FTB and PROM (groups C and D) (Figure 3).

## Discussion

### Placental Function and Preterm Birth

PTB plays a significant adverse impact on the increased mortality and mobility of preterm-born neonates. The etiology of sPTB is multi-factorial; however, placental dysfunction has been identified as the leading cause of premature birth due to it pivotal role between the fetus and mother during pregnancy (Audette and Kingdom, 2018). As a result of the pathophysiological changes in placental dysfunction, including poor trophoblast uterine invasion and impaired transformation of the uterine spiral arteries to high capacity and low impedance vessels, which eventually leads to lower blood flow to the placenta (Ilekis et al., 2016; Cuffe et al., 2017), the placenta was unable to sustain fetal development requirements and thus preterm birth occurred. Searches for molecular markers to predict preterm birth have been conducted mainly in the maternal blood due to its richness of information and easy accessibility. It has been reported that PP-13 and PAPP-A are good predictors of preterm birth (Stout et al., 2013). Elevated maternal serum–soluble fms-like tryrosine 1 (sFlt1), inflammation marker [cysteine-rich protein (CRP)], and PIGF are associated with preterm birth (Bastek et al., 2011; Straughen et al., 2012). However, these markers have little clinical use for the prediction of preterm birth or for understanding the pathophysiology of placental dysfunction. Therefore, we conducted an unbiased proteomic analysis of the placenta in preterm birth compared to in full-term controls, followed by various comparisons to generate differentially expressed proteins specific to one single variation. Such comparisons include group B vs. group C, for example, in which the outcome of differentially expressed proteins was influenced only by premature rupture of fetal membrane in PPROM (B) vs. FTB without rupture of membrane (C). In addition, such comparisons may reduce non-specific noise, such as the comparison of group A vs. group D, in which the differentially expressed proteins would likely have resulted from the premature labor that was related to muscle contraction of uterine but not to rupture of membrane.

### Proteins Involved in Infection and Inflammation

Based on molecular function, the top-scoring proteins differentially expressed in fetal membranes of sPTB have been clustered into five groups *via* their involvement in pathophysiological pathways (Table 3). The main pathophysiological changes in the fetal membrane from preterm birth were inflammation. Numerous studies indicated that placental intrauterine infection was strongly associated with sPTL or PPROM (Salafia et al., 1991; Helmig et al., 2002; Morgan, 2016; Pugni et al., 2016; Chisholm et al., 2018).

A study in humans demonstrated that HPGD expression in preterm-labor placenta tissues was decreased in comparison with a full-term labor group (He et al., 2015). HPGD is a member of the short-chain non-metalloenzyme alcohol dehydrogenases protein family and is responsible for the degradation of prostaglandins, hormones that modulate the inflammatory response (Aoki and Narumiya, 2012; Seo and Oh, 2017). Another study in an animal model detected high expression levels of HPGD at the beginning and at normal term of pregnancy, indicating that HPGD may play a role during the establishment and termination of gestation (von Hof et al., 2017). Our data are consistent with previously findings. The lower level of HPGD found in fetal membranes suggested a higher level of inflammatory states in the preterm group (sPTL + PPROM), possibly through negative regulation of inflammatory molecule prostaglandins.

PTPRC is a receptor-type protein tyrosine phosphatase that regulates cell growth, differentiation, and mitosis. PTPRC is essential to regulate T- and B-cell antigen receptor signaling by direct interaction with antigen receptor complexes or by activating Src family kinases (Pike and Tremblay, 2013). PTPRC also regulates cytokine receptor signaling by suppressing JAK kinase (Porcu et al., 2012). It has been reported that PTPRC is dysregulated in human miscarriage (Lorenzi et al., 2012). Increased levels of PTPRC in the fetal membranes of sPTB indicated activation of T- and B-cell antigen receptor signaling and suggested that sPTB may share a common pathophysiological mechanism with miscarriage.

Macrophage activation (CAPG, CD14, and CD163): CAPG is a member of the gelsolin/villin family of actin-regulatory proteins. CAPG reversibly blocks the barbed ends of F-actin filaments in a Ca^2+^-dependent manner, thus capping the barbed ends of actin filaments and controlling actin-based motility of macrophages (Young et al., 1994). CD14 is a surface protein preferentially expressed on macrophages or monocytes. It mediates the innate immune response to bacterial lipopolysaccharide (Mogensen, 2009). A study in an animal model indicated that increased expression of  TLR2 and CD14 was correlated with urea plasma parvum–induced fetal inflammatory response syndrome-like pathology (Allam et al., 2014). CD163 is a member of the scavenger receptor cysteine-rich superfamily and functions as an innate immune sensor for bacteria and an inducer of local inflammation (Fabriek et al., 2009). High levels of CD163 are associated with an increased risk of preterm delivery in pregnant women (Vogel et al., 2005). The detection of CAPG, CD14, and CD163 suggested the activation of macrophage or monocyte, even though we do not know what pathogen causes are.

### Extracellular Matrix, Proteolysis, and Cell Adhesion

COL4A1, LAMA2, FBLN2, and APMAP are the ECM proteins. COL4A1 is an integral component of all basement membranes; it not only provides structural support, regulating adhesion, migration, and survival of cells, but also plays a key role in early placentation by modeling trophoblast cell invasion to remodel maternal spiral arteries and ensure sufficient blood flow to the developing fetus (Oefner et al., 2015). LAMA2 is a major component of the basement membrane and mediates the attachment and migration of cells into tissues during embryonic development by interacting with other ECMs. FBLN2 is an ECM protein belonging to the fibulin family. APMAP is a novel regulator of ECM components that may serve as a potential target to mitigate obesity-associated insulin resistance (Pessentheiner et al., 2017). The exact functions of LAMA2, FBLN2, and APMAP in the placenta are not clear; the expression level changes may indicate ECM degradation in PPROM and PROM. CSRP1 is a membrane of the CRP family, which may regulate cellular development and differentiation.

MMP9, ELANE, and chymotrypsin C (CTSG) are the proteins involved in proteolysis. MMP9 has been reported to break down the ECM, such as type IV and V collagens. MMP9 is mainly expressed in amnion epithelia, chorion leave trophoblast, decidua parietalis, and placental syncytiotrophoblasts. The expression level of MMP9 was increased in fetal membranes from preterm and term labor as compared to non-labors (Xu et al., 2002). Moreover, fetuses with PPROM have higher concentrations of MMP-9 than those with preterm labor with intact membrane, indicating the pathogenic role of MMP-9 during a rupture of fetal membrane in sPTB (Romero et al., 2002). The level of MMP-9 has been used as a risk factor for preterm births (Di Ferdinando et al., 2010; Sorokin, 2010). In agreement with published data, an increase in MMP-9 expression may contribute to degradation of the ECM in the fetal membrane and in placentas, thus initiating sPTB. ELANE, a multifunctional serine protease stored in azurophilic granules of mature neutrophils, is able to degrade the ECM of connective tissue during an inflammatory process. It has been implicated that PPROM, microbial invasion of the amniotic cavity, and parturition at term and preterm are associated with a significant increase in the concentration of ELANE in the amniotic fluid. Another study suggested that ELANE levels in amniotic fluid may serve as a useful marker for predicting the duration of continued pregnancy after cervical cerclage (Hatakeyama et al., 2016). CTSG is a member of the peptidase S1 protein family in azurophilic granules of neutrophilic polymorphonuclear leukocytes. CTSG has a cleavage specificity similar to chymotrypsin C and may involve connective tissue remodeling at the site of inflammation. It has been shown that intra-amniotic inflammation (IAI) is associated with increased CTSG concentration in the amniotic fluid in PPROM (Musilova et al., 2017). It has been reported that the ubiquitin–proteasome–collagen (CUP) pathway is involved in collagen degradation in PPROM (Zhao et al., 2017). In addition, the CUP pathway was epigenetically regulated by IncRNA in PROM and PPROM (Luo et al., 2015). Even though we did not identify proteins belonging to the CUP pathway, we did identify the most significant proteolytic enzymes associated with PPROM and PTB, mainly MMP-9, serine protease (ELANE), and CTSG.

Our proteome data detected over-expression of cell adhesion molecules ICAM and ITGAM in sPTB. ICAM-1 is a cell surface glycoprotein expressed on endothelial cells and cells of the immune system (Egal et al., 2014). It has been reported that ICAM-1 was overexpressed in villous trophoblasts during placental infection (Juliano et al., 2006). Another study indicated that expression of ICAM-1 by the human choriodecidua was elevated with preterm birth, together with increased leukocyte infiltration (Marvin et al., 1999). ITGAM plays an important role in the adherence of neutrophils and monocytes to stimulated endothelium. The level of ITGAM was significantly elevated in the amniotic fluid of women with preterm labor with IAI compared without IAI (Romero et al., 2010). Again, increased levels of ICAM-1 and ITGAM suggest activation of white blood cells and immune response in preterm birth placenta membrane.

### Oxidative Stress Proteins MPO, LMNB1, and LMNB2

MPO is a heme protein synthesized and released from activated monocytes and neutrophils. Traditionally, it was described as a microbicidal enzyme. New evidence indicates that MPO generates hypochlorite-modified proteins, activates metalloproteinase (Prokopenko et al., 2002), and oxidatively consumes endothelium-derived nitric oxide in humans during normal pregnancy as well as during pathophysiologic processes (pre-eclampsia) (Hammer et al., 2001; Gandley et al., 2008). Higher cord blood levels of MPO are associated with preterm delivery (Yang et al., 2004). LMNB1 and LMNB2 are B-type nuclear lamin located in the inner nuclear membrane and play an important role in nuclear stability, chromatin structure, and gene expression. Overexpression of LMNB1 was found through a mitochondrial reactive oxygen species (ROS) *in vitro* model to increase the proliferation rate and to delay the onset of senescence (Shimi et al., 2011). Oxidative stress was higher and antioxidant enzymes were lower in PPROM compared with sPTB (Dutta et al., 2016). Our data suggest a higher level of oxidative stress, which may induce premature cellular senescence, inflammation, and proteolysis, eventually leading to membrane rupture and PPROM.

### Glycolysis, Fatty Acid Synthesis

Differentially expressed proteins involved in glycolysis and FA synthesis were down-regulated in the fetal membranes of sPTB (Table 3). PKM, a catalytic enzyme involved in the last step of glycolysis, is responsible for dephosphorylating phosphoenolpyruvate to pyruvate and producing ATP under hypoxic conditions. PKM is also involved in angiogenesis in embryo development. It has been reported that the expression of PKM is higher in pre-eclampsia at delivery than in normal pregnancy (Bahr et al., 2014). ADPGK is a rate-limiting enzyme within the first step of glycolysis; it catalyzes the phosphorylation of D-glucose to D-glucose 6-phosphate by using ADP as the phosphate donor. GAPDH is an enzyme responsible for glyceraldehyde dehydration in the process of glycolysis. The placenta is a main source of high lactate levels during gestation. In later gestation, the concentration of lactate can reach 10 mmol/l, as compared to 1–2 mmol/l in the newborn and 0.5 mmol/l in maternal plasma. Fatty acids are essential substances for the construction of cell membrane and development of the nervous system. The disruption of FAs’ metabolism in the maternal-placental interface would result in malnutrition of the fetus and in preterm birth (Bobinski and Mikulska, 2015). Blood levels of FAs are 20 times lower in the fetal circulation than in newborns’ circulation (Makinde et al., 1998). This indicates that glucose/lactate provides the main energy source in fetal development. Typically, lactate is described as a waste product catalyzed by LDH under anaerobic metabolism. A metabolism study in various cell types indicated a model whereby lactate generated in the cytosol compartment can be oxidized into pyruvate in mitochondria by LDH; pyruvate is subsequently transported into the inner membrane of the matrix and is then oxidized to acetyl CoA by pyruvate dehydrogenate (Kane, 2014). Acetyl CoA can then be fed into the TCA cycle and maintains mitochondrial function. Our data showed an increase in the level of ADPGK in the first step of glycolysis and a reduced level of PKM in the last step of glycolysis, suggesting an imbalance of glycolysis and the generation of less pyruvate through the conventional glycolysis pathway. To maintain metabolic functionality, a lactate oxidation process in mitochondria to produce pyruvate to sustain the TCA cycle is preferred. Reduction of the proteins involved in glycolysis and fatty acid synthesis may suggest that energy synthesis might be reduced in the fetal membrane tissues in sPTB. To confirm this, further investigation is needed to understand better the role of lactate’s and FAs’ metabolism in sPTB.

### Tissue Specificity

Differential expression patterns of GAPDH were documented between the fetal membranes and placental villi. As shown in the right panel of Figure 3, GAPDH was found to be down-regulated in premature birth groups A and B, as compared to full-term control groups C and D with (PROM) or without (FTB) rupture of membrane in samples of fetal membrane. This finding is in agreement with the results generated from a discovery study with MS/MS. However, in the tissue of placental villi, Western blot for validation showed up-regulation, compared to FTB, which resulted in no significant change in placenta specimens between A + B vs. C + D. GAPDH is an enzyme involved in anaerobic energy metabolism through glycolysis. We speculated that the glycolysis pathway may be affected in fetal membrane and weakened the structure of fetal membrane but not in placental villi. To confirm this, further investigation should be performed to provide biochemical evidence.

## Conclusion

By applying a proteomic approach, along with validation of proteomic results with Western blots, to study sPTL and PPROM with the capacity of distinguishing between the sPTL and PPROM groups, and of distinguishing the premature groups sPTL and PPROM from the mature groups FTB and PROM, we demonstrated a unique signature for each of the conditions, which is the strength this study. In addition, our proteomic data provide systemic insights into pathophysiological changes in the fetal membranes on the molecular level. Our data support the theory of inflammation/infection in sPTL and PPROM, even though we have not identified the source of infection. Inflammation increased the oxidative stress–triggered pyrolytic process that changes ECM structure, eventually rupturing the placental membrane and resulting in preterm birth. Metabolic function was also altered, in particular, imbalanced glycolysis and unconventional lactate oxidation, which may be associated with preterm birth. We are also aware of the limitation that applying the proteomic approach to identifying the differentially expressed protein(s) that are associated with clinical features and to identify biomarker(s) for the disease condition could be influenced by post-translational modification. Therefore, a larger sample size will need to be used for validation. In fact, studies on the inflammation-triggered proteolysis of ECM has been undertaken to verify a key pathogenic pathway for sPTB.

## Data Availability Statement

The datasets presented in this study can be found in online repositories. The names of the repository/repositories and accession number(s) can be found in the article/supplementary material.

## Ethics Statement

The study was approved by the Hospital Ethics Committee of Sanya Maternity and Child Care Hospital. Written informed consent was obtained from pregnant women to permit the use of placentas in research studies.

## Author Contributions

JP, XT, and HH contributed to the sample collection, processing and preparation, data acquisition, and laboratory work. NZ contributed to, and was responsible for, conceptual research design, initiating and coordinating the studies and experiments, data analysis, and drafting, finalizing, and submitting the manuscript. All authors contributed to the article and approved the submitted version.

## Conflict of Interest

The authors declare that the research was conducted in the absence of any commercial or financial relationships that could be construed as a potential conflict of interest.
